# Exploration of larvicidal activity of *Vernonia anthelmintica* (L.) wild seed crude extracts in different solvents against malaria (*Anopheles stephensi*) and dengue (*Aedes aegypti*) vectors

**DOI:** 10.1186/1475-2875-11-S1-P46

**Published:** 2012-10-15

**Authors:** Alina Hellert, Gaurav Sharma, Kaushal Kumar, Veena Agrawal

**Affiliations:** 1 Plantphysiology Department, University of Bayreuth, Bayreuth, 95440, Germany; 2 Department of Botany, University of Delhi, Delhi, 110007, India; 3 Centre of Medical Entomology & Vector Management, National Centre for Disease Control, Delhi, 110054, India

## Background

A large part of the population in the world is affected by one or more vector-borne diseases. The most effective way to prevent such diseases is to control the vectors [1]. Plant based insecticides are one of the best alternatives for the hazardous chemicals [2]. Leaves and fruits of *Vernonia anthelmintica* have been reported to have larvicidal properties against malaria vector [3]. In this study the larvicidal activity of the seeds of *V. anthelmintica* has been investigated for the first time.

## Materials and methods

In this study, larvicidal activity of crude ethanol, hexane, acetone chloroform and methanol extracts of the seeds of *V. anthelmintica* were tested against late Ill/early IV stages larvae of malaria (*Anopheles stephensi* (Liston)) and dengue (*Aedes aegypti* (Linnaeus)) vectors [4], [5].

## Results

All tested extracts showed strong larvicidal activity against the both vectors. The most effective extract against malaria vector was ethanol followed by chloroform and methanol extracts (LC50 1.95, 3.535 and 3.974 ppm; LC90 10.49, 18.325 and 15.979 ppm). Whereas in case of dengue vector chloroform was most effective (LC50 2.76 and LC90 14.01) followed by methanol and ethanol extracts LC50 3.395 and 3.461 ppm (LC90 12.95 and 12.804 ppm) (Table 1 and 2).

**Table 1:** Larvicidal activity of different extracts of *V.anthelmintica* seeds against Late III/Early VI instar larvae of *A. stephensi.* No mortality was observed in the controls

Solvent used for extraction	Concentration of crude extract (ppm)	Mortality (%)	LC_50_ (ppm) (LCL - UCL)	LC_90_(ppm)(LCL-UCL)	***X*** **^2a^**
Ethanol	50	100	1.945	10.492	5.220
	25	99	(1.488 - 2.392)	(8.479 - 13.837)	
	12.5	92			
	6.25	75			
	3.13	63			
	1.56	48			
Hexane	50	72	22.452	147.764	2.725
	25	48	(18.692 - 27.831)	(100.792 - 248.774)	
	12.5	36			
	6.25	23			
	3.13	9			
	1.56	2			
Acetone	50	83	9.124	74.713	2.231
	25	72	(7.606 - 10.957)	(53.036 - 118.306)	
	12.5	62			
	6.25	41			
	3.13	28			
	1.56	11			
Chloroform	50	100	3.535	18.324	14.713**
	25	98	(1.880 - 5.401)	(10.974 - 52.048)	
	12.5	79			
	6.25	57			
	3.13	45			
	1.56	34			
Methanol	50	100	3.974	15.979	8.367
	25	97	(3.413 - 4.567)	(13.198 - 20.310)	
	12.5	87			
	6.25	56			
	3.13	40			
	1.56	25			

LC_50_ Lethal concentration that kills 50% of exposed larvae; LC_90_ Lethal concentration that kills 90% of exposed larvae; LCL Lower confidence limits; UCL Upper confidence limits; *χ*^2^Chi-square; ** Significant at *P* <0.01; ^a^ degree of freedom 4

**Table 2:** Larvicidal activity of different extracts of *V.anthelmintica* seeds against Late III/Early VI instar larvae of *A.aegypti.* No mortality was observed in the control

Solvent used for extraction	Concentration of crude extract (ppm)	Mortality (%)	LC_50_ (ppm) (LCL - UCL)	LC_90_(ppm)(LCL-UCL)	***X*** **^2a^**
Ethanol	50	100	3.461	12.804	15.230**
	25	99	(2.088 - 5.012)	(8.267 - 30.435)	
	12.5	94			
	6.25	59			
	3.13	43			
	1.56	29			
Hexane	50	83	14.503	105.282	5.062
	25	61	(12.174 - 17.563)	(73.710 - 169.855)	
	12.5	40			
	6.25	30			
	3.13	21			
	1.56	6			
Acetone	50	93	5.563	47.350	1.837
	25	80	(4.537 - 6.699)	(34.556 - 72.194)	
	12.5	66			
	6.25	51			
	3.13	40			
	1.56	22			
Chloroform	50	99	2.761	14.009	3.024
	25	98	(2.244 - 3.285)	(11.338 - 18.379)	
	12.5	88			
	6.25	69			
	3.13	53			
	1.56	36			
Methanol	50	100	3.393	12.945	9.720*
	25	100	(2.301 - 4.584)	(8.950 - 24.277)	
	12.5	89			
	6.25	65			
	3.13	42			
	1.56	30			

LC_50_ Lethal concentration that kills 50% of exposed larvae; LC_90_ Lethal concentration that kills 90% of exposed larvae; LCL Lower confidence limits; UCL Upper confidence limits; χ^2^ Chi-square;* Significant at *P* <0.05;** Significant at *P* <0.01; ^a^ degree of freedom 4

## Conclusion

This is the first report of cent percent mortality against the vectors of malaria and dengue using minimal doses of the seed extracts of *V. anthelmintica.* Further work for the isolation and characterization of larvicidal compounds is in progress.
